# The International Conference on Intelligent Biology and Medicine (ICIBM) 2020: Data-driven analytics in biomedical genomics

**DOI:** 10.1186/s12920-020-00833-7

**Published:** 2020-12-28

**Authors:** Xinghua Shi, Zhongming Zhao, Kai Wang, Li Shen

**Affiliations:** 1 Department of Computer and Information Sciences, College of Science and Technology, Temple University, Philadelphia, PA 19122 USA; 2 Center for Precision Health, School of Biomedical Informatics, The University of Texas Health Science Center at Houston, Houston, TX 77030 USA; 3 Raymond G. Perelman Center for Cellular and Molecular Therapeutics, Children’s Hospital of Philadelphia, Philadelphia, PA 19104 USA; 4 Department of Pathology and Laboratory Medicine, University of Pennsylvania, Philadelphia, PA 19104 USA; 5 Department of Biostatistics, Epidemiology and Informatics, Perelman School of Medicine, University of Pennsylvania, Philadelphia, PA 19104 USA

**Keywords:** Genomics, Gene regulation, Integrative analysis, Human diseases

## Abstract

This editorial summarizes eight research articles included in this supplement issue for the 2020 International Conference on Intelligent Biology and Medicine (ICIBM 2020) conference, that was held on August 9-10, 2020 (virtual conference), with a topic on data-driven analytics in biomedical genomics. These articles cover a wide range of topics in medical genomics that focus on integrative analysis of genomics data together with other types of data toward understanding complex human diseases, including cancer. With the growing importance of data analytics in biomedical science, we expect this collection of research articles provides scientific discussions in this direction.

## Introduction

The 2020 International Conference on Intelligent Biology and Medicine (ICIBM 2020), the official conference of the International Association for Intelligent Biology and Medicine (IAIBM), was held virtually from August 9th to 10th, 2020 due to the COVID-19 pandemic. Established in 2012, the ICIBM conference has grown as a venue to cultivate interdisciplinary research and education at the intersection of bioinformatics, intelligent computing, systems biology, and medical informatics. The first virtual conference of ICIBM (ICIBM 2020) had approximately 300 attendees, with 41 oral presentations scheduled in four live sessions selected from 75 original submissions. With rigorous review for ICIBM 2020 followed by second-round reviews for journal submissions, eight high-quality manuscripts were selected to be published in the ICIBM 2020 BMC Medical Genomics special issue.

## Summaries of manuscripts in this issue

This supplementary issue includes eight manuscripts that cover a variety of topics in medical genomics, with a focus on data-driven analytics in biomedical genomics research. Here we summarize the contribution of each of these eight manuscripts.

In the manuscript titled “A pan-kidney cancer study identifies subtype specific perturbations on pathways with potential drivers in renal cell carcinoma”, Zhan et al. [1] introduced a study that utilized pathway analysis to identify biological pathways that are common or specific to different subtypes of renal cell carcinoma (RCC). They first conducted differential gene expression analysis to identify pathway perturbations in different RCC subtypes using RAN-sequencing data extracted from The Cancer Genome Atlas (TCGA). For subtype-specific pathways, they further assessed potential upstream regulators of these pathways and evaluated the relationship between subtype-specific pathways and disease outcomes in RCC. This study provided hypotheses that alterations of upstream regulators affect perturbations of downstream pathways in different subtypes of RCC, may introduce differences in cancer initialization and prognosis.

In the study of “Pinpointing miRNA and genes enrichment over trait-relevant tissue network in Genome-wide Association Studies”, Li et al. [2] combined the analysis of microRNA expression, GWAS signals, and miRNA-gene networks to identify miRNAs and their targeted genes that harbor genetic variants in different tissues. With an aim of identifying functional GWAS SNPs that may change the binding affinity of miRNAs and genes, they used several bioinformatics tools to annotate GWAS SNPs that are located with miRNA targeted genes in relevant tissues. As an example, they performed GO analysis on genes that harbor GWAS SNPs in 3’UTR regions of genes that are targeted by miRNAs for the trait of primary biliary cirrhosis.

In the manuscript titled “Characterization of genome-wide association study data reveals spatiotemporal heterogeneity of mental disorders”, Dai et al. [3] presented an integrative analysis framework that coupled GWAS summary statistics with spatiotemporal gene co-expression modules to investigate risk genes and their co-expression partners in mental disorders. Applying this framework to the BrainSpan data, this study evaluated genetic factors underlying critical spatiotemporal points in brain development for five psychiatric disorders including schizophrenia, bipolar, major depression, attention deficit-hyperactivity disorder, and autism. Their results indicated that although these mental disorders shared some disease genes, the genetic predisposition to these disorders are largely specific to each disorder. This study demonstrated the integration of RNA-seq data with GWAS statistics to identify functional variants that are associated with human traits and diseases.

In “Network-based Drug Sensitivity Prediction”, Ahmed et al. [4] explored network-based methods for drug sensitivity prediction, including a newly developed method that first used gene coexpression networks to extract representative features and then used graph-based neural network models for drug response prediction. Applying this method to RNA-seq data in non-small cell lung cancer cell lines with treatments of 50 different drugs, this study demonstrated that combining network-based feature selection with graph-based prediction methods, they were able to improve the performance of predicting drug sensitivity and response. These findings thus pointed to a direction where graph-based machine learning methods including emerging graph-based deep neural networks have great potential to integrate high-dimensional omics data for drug response prediction.

In the study of “Differential alternative splicing (AS) between hepatocellular carcinoma with normal and elevated serum alpha-fetoprotein”, Jin et al. [5] reported an investigation of differential AS events in hepatocellular carcinoma (HCC) patients with high and normal serum alpha-fetoprotein (AFP) levels. Using RNA-seq data extracted from TCGA, they identified a set of AS events for genes that are enriched for cell migration or proliferation, and some of these genes are associated with gender and vascular invasion. These findings point to the critical roles that AS may play in regulating transcription differences for modulating AFP levels in HCC patients.

In the manuscript titled “Integrative analysis of histopathological images and chromatin accessibility data for estrogen receptor-positive breast cancer”, Xu et al. [6] provided an integrative analysis of histopathological images and genomic data (ATAC-seq data and matched RNA-seq data) to pinpoint regulatory regions associated with epithelial tissue proportion in estrogen receptor (ER) positive breast cancer. They started with using convolutional neural network (CNN)-based models to identify epithelial and stromal tissues from whole-slide images of patients and then calculated epithelial tissue proportions utilizing data from hemotoxin and eosin (H&E) stained slides. They then computed correlations between epithelial tissue proportion and the chromatin accessibility for open chromatin regions detected in the ATAC-seq data to identify open chromatin regions that are significantly associated with epithelial tissue proportion. Consequently, they evaluated target genes of these regions and found that these genes were enriched for oncogenes and relevant pathways. This integrative analysis pipeline can thus be applied to prioritize candidate gene regulatory regions and their target genes to shed light on the molecular mechanisms of breast cancer and many other diseases.

In the manuscript titled “The circular RNA expression profile in ovarian serous cystadenocarcinoma reveals a complex circRNA-miRNA regulatory network”, Zhuang et al. [7] studied the landscape and differential expression of circular RNAs (circRNAs) and its co-expression with mRNAs and microRNAs in ovarian serous cystadenocarcinoma (OSC), the most common type of ovarian cancer. Using RNA sequencing of specimens taken from tumor and normal tissues in patients, they identified 15,092 circRNAs including novel ones in tumor and normal tissues. The expression of these circRNAs differ with tumor and normal tissues, with more circRNAs downregulated in tumor tissues. By comparing the expression of circRNAs and their homologous mRNAs, this study suggested a strong relationship between circRNAs and mRNAs, and some cirRNAs serving as potential biomarkers for this disease.

In the manuscript titled “Conditional transcriptional relationships may serve as cancer prognostic markers”, Yu et al. [8] incorporated differential gene correlation analysis with survival analysis to identify conditional transcriptional relationships as potential prognostic markers for cancer. In this study, the authors leveraged recent advances in differential coexpression (DC) methods for modeling correlations between gene pairs that significantly differ between normal and tumor tissues respectively in 13 cancer types with RNA-sequencing data downloaded from TCGA. After utilizing the Correlation by Individual Level Product (CILP) approach to identify differentially coexpressed links among gene pairs, they conducted survival analysis on ten cancer types with data available. Their results showed that these identified conditional transcriptional relationships between genes may serve as candidate prognostic biomarkers for human cancers.

## Conclusion

This supplementary issue includes a collection of eight manuscripts that are focused on various perspectives of integrating data across multiple data types (e.g. genomics, imaging or interaction data), to understand the etiology, prognostics and progression of diseases. With the emerging big data with regards to its volume, velocity, and variety in biomedical science, we anticipate integrative analytics will significantly propel future biomedical advances in realizing the full potentials of big data in biomedicine.
